# Phasic Left Atrial Function in Cancer Patients Before Initiation of Anti-Cancer Therapy

**DOI:** 10.3390/jcm8040421

**Published:** 2019-03-27

**Authors:** Marijana Tadic, Martin Genger, Cesare Cuspidi, Evgeny Belyavskiy, Athanasios Frydas, Aleksandar Dordevic, Daniel A. Morris, Jakob Völkl, Abdul Shokor Parwani, Burkert Pieske, Sabine Haßfeld

**Affiliations:** 1 Department of Internal Medicine and Cardiology, Charité-University-Medicine Berlin, Campus Virchow Klinikum (CVK), 13353 Berlin, Germany; martin.genger@charite.com (M.G.); Evgeny.Belyavskiy@charite.com (E.B.); Athanasios.Frydas@charite.com (A.F.); Aleksandar.Dordevic@charite.com (A.D.); daniel.morris@charite.com (D.A.M.); Jakob.Voelkl@charite.com (J.V.); Abdul.Parwani@charite.com (A.S.P.); burjert.pieske@charite.com (B.P.); Sabine.hassfeld@charite.com (S.H.); 2 Clinical Research Unit, University of Milan-Bicocca and Istituto Auxologico Italiano, 20821 Meda, Italy; cesare.cuspidi@inimib.com; 3 Deutsches Zentrum für Herz-Kreislauf-Forschung, Standort Berlin/Charité, 13353 Berlin, Germany; 4 Department of Cardiology, Deutsches Herzzentrum Berlin, 13353 Berlin, Germany

**Keywords:** cancer, left atrium, strain, phasic function

## Abstract

We aimed to explore left atrial (LA) remodeling in the patients with solid cancer before initiation of chemo- or radiotherapy. This retrospective investigation included 92 chemo- and radiotherapy-naive cancer patients and 40 age- and gender-matched controls with a similar cardiovascular risk profile as the cancer group. All participants underwent comprehensive echocardiographic examination before the start of chemo- or radiotherapy. LA phasic function was evaluated in volumetric and strain method. Indexed minimal and pre-A LA volumes were significantly higher in the cancer patients. Total and passive LA emptying fraction (EF) were significantly lower, whereas active LAEF was significantly higher in the cancer patients. LA total longitudinal strain was significantly lower in the cancer patients. Strain rate analysis of LA phasic function showed that LA function during systole and early diastole was reduced in the cancer group, while it was increased during late diastole. These findings indicated that LA reservoir and conduit functions, assessed with LA volumetric and strain analysis, were deteriorated in the cancer group. On the other hand, LA booster pump function was elevated in the cancer group in comparison with the controls. In the whole population, cancer was associated with reduced LA total longitudinal strain independently of age, gender, BMI, LV hypertrophy, E/e’ ratio, diabetes, and hypertension. LA phasic function was impaired in the chemo- and radiotherapy-naive cancer patients in comparison with the control group. Cancer, LV hypertrophy, and hypertension were associated with reduced LA longitudinal strain independently of other important clinical parameters.

## 1. Introduction

Left ventricular (LV) remodeling in the treated cancer patients has been confirmed in the large number of studies [1,2,3], and particularly important is the incremental predictive value of LV changes in the cancer patients [3]. Our study group recently showed that left and right ventricular functional impairment, particularly in strain, was present in the cancer patients before anti-cancer therapy [4,5]. However, LA remodeling in these patients is still a matter of debate [2,6]. The vast majority of investigations was focused on the cancer patients during and after chemotherapy or radiotherapy and none of them investigated LA phasic function in chemo- and radiotherapy-naive patients. 

Recently published studies showed significant dilatation and reduction in LA contractile function in patients after chemotherapy [6,7,8]. The authors claimed that LA dilatation during chemotherapy predicted cardiotoxicity development in HER2-positive breast cancer patients [8]. A recent investigation showed that it was not baseline LAV that indexed for BSA (LAVI), but that increment of LAVI during trastuzumab was associated with cardiotoxicity [9]. There are still no data regarding LA structural and functional remodeling in the cancer patients before initiation of chemo- or radiotherapy. Considering the fact that LA function has an important implication with respect to outcomes in general population [10], the evaluation of LA phasic function in the cancer patients before anti-cancer treatment could be of importance due to the potential to discriminate LA remodeling induced by chemo- or radiotherapy from LA dysfunction that exists before treatment. Furthermore, LA enlargement and dysfunction before treatment initiation might be related with higher risk of cardiotoxicity in the later course of disease when therapy is applied. This hypothesis represents the rationale for this study.

The primary aim of this investigation was to evaluate LA phasic function in the chemo- and radiotherapy-naive cancer patients meaning before starting any anti-cancer treatment. The secondary aim was to explore the association between cancer and LA total longitudinal strain.

## 2. Methodology

### 2.1. Population

This is a retrospective study that involved 92 consecutive patients with solid cancer who were referred to echocardiographic assessment before initiation of chemotherapy or radiotherapy in the period between January 2016 and July 2017. The inclusion criteria were normal LV ejection fraction (EF > 50%) and absence of any chemotherapy or radiotherapy. Additionally, 40 subjects of similar age and sex distribution with the same profile of cardiovascular risk factors (arterial hypertension, diabetes, and smoking) as the cancer patients and who were referred to our echocardiography laboratory in the same period as the cancer patients were retrospectively included as the control group. The subjects in the control group were referred to the echocardiographic department due to regular check-up, an innocent heart murmur, or as a part of investigation in hypertensive patients. Patients with cancer metastasis, symptoms or signs of coronary artery disease, more than mild valvular heart disease, heart failure, atrial fibrillation, congenital heart disease, or liver or kidney failure were excluded from this study. Patients with inadequate echocardiographic images of the LA were not included (n = 18). 

All the patients underwent anthropometric measurement (height, weight). Body mass index (BMI) and body surface area (BSA) were calculated for each patient. Data regarding blood pressure values, hypertension, diabetes, smoking, and other comorbidities were obtained from the electronic medical records. According to the rules of the Ethics Committee of our clinic, a retrograde study does not require approval if the investigators only use the existing data, without follow-up or any further contact with the study participants.

### 2.2. Echocardiography

An EPIQ 7 (Philips Healthcare, Germany) ultrasound machine was used for echocardiographic examinations of all the participants. The frame rate ranged between 60 and 100 Hz. Reported values of all 2DE parameters were obtained as the average value of three consecutive cardiac cycles. LV diameters and septum and relative wall thickness were measured according to the current recommendations [11]. LV ejection fraction (EF) was calculated by using the biplane method. LV mass (LVM) was calculated by using the corrected ASE method and indexed for BSA [11]. Left ventricular hypertrophy was defined according to the latest guidelines (LV mass index > 95 g/m^2^ for women and > 115 g/m^2^ for men) [11].

The apical four-chamber view was used for the assessment of transmitral LV flow and deceleration time by the pulsed-wave Doppler [12]. Tissue Doppler imaging was used to obtain LV myocardial velocities in the apical four-chamber view, with a sample volume placed at the septal and lateral segments of the mitral annulus during early diastole (e’). The average of the peak early diastolic relaxation velocity (e’) of the septal and lateral mitral annulus was calculated, and the E/e´ ratio was computed. Mitral E/A and E/e’ ratios were used for the assessment of LV diastolic function [12].

### 2.3. DE Assessment of Left Atrial Volumes and Function

**Figure 1:**
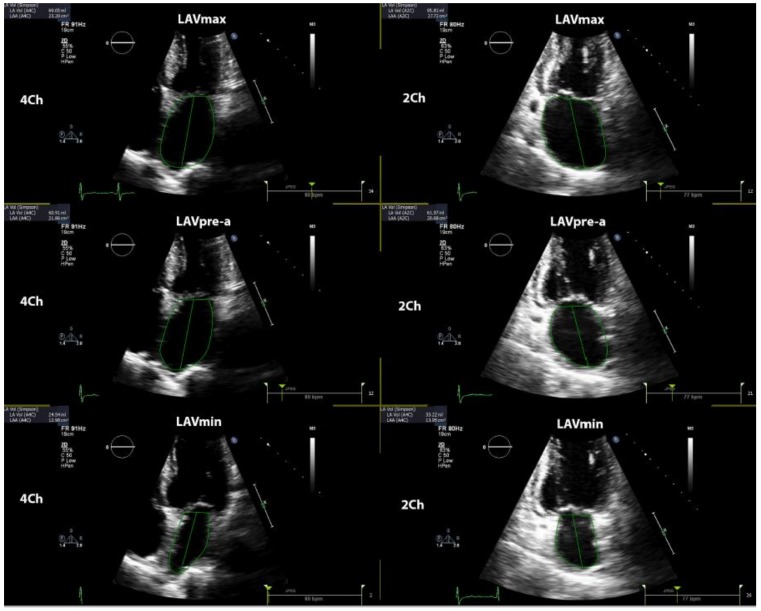
Shows the methodology of determination of left atrial volumes. LAV_max_—maximal left atrial volume; LAV_min_—minimal left atrial volume; LAV_pre-a_—left atrial volume before atrial contraction.

LA phasic function was determined by volumetric and strain analysis. LA volumes were obtained in three various parts of the cardiac cycle: maximal LA volume was calculated just before the mitral valve opening, pre-A (pre atrial contraction) LA volume was evaluated at the beginning of atrial systole (peak of P wave in ECG), and minimal LA volume was assessed at the mitral valve closure. All LA volumes were determined according to the biplane method in four- and two-chamber views and all the values were indexed for BSA. The total emptying volume (LA reservoir function parameter) was calculated as the difference between maximum and minimum LA volume; passive emptying volume (LA conduit function parameter) was computed as the difference between maximum and pre-A LA volume; and active emptying volume (LA booster function parameter) was calculated as the difference between pre-A and minimum LA volume [13]. Figure 1 shows the methodology of determination of LA volumes in the current study. Total emptying fraction (EF) was calculated as the ratio between total emptying volume and maximum LA volume; passive EF was computed as the ratio between passive and maximum; and active EF was evaluated as the proportion between active and pre-A LA volume.

**Figure 2:**
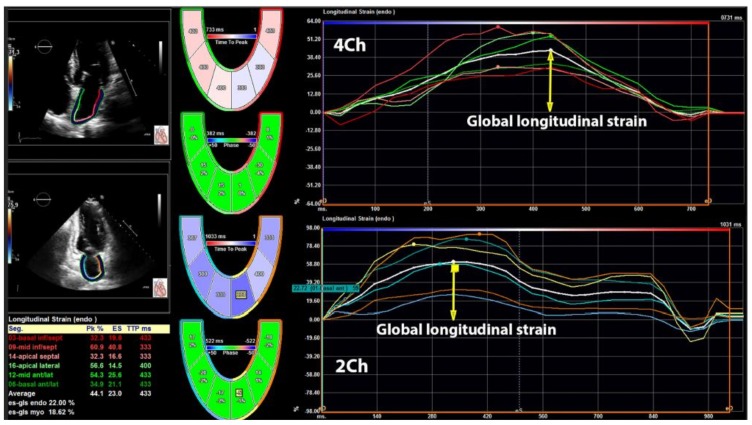
Illustrates the methodology of left atrial longitudinal strain evaluation in four- and two-chamber views.

**Figure 3:**
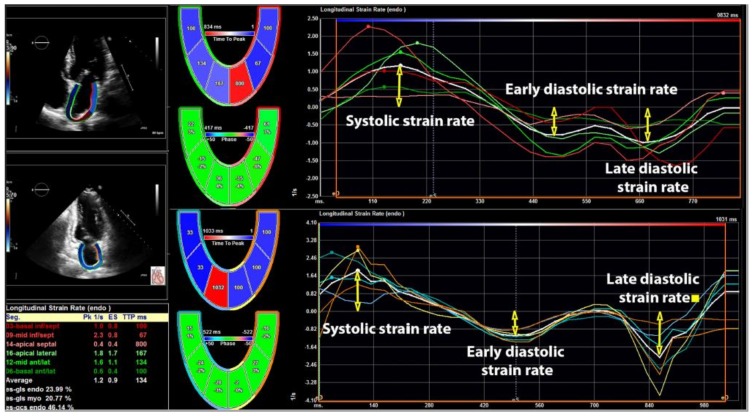
Assessment of left atrial longitudinal strain rates in four- and two-chamber views.

The 2DE strain analysis was performed in the apical four- and two-chamber views [14] by commercially available TomTec software (Unterschleissheim, Germany). The LA endocardium was manually traced and average longitudinal strain curve was automatically provided by the software. The software provided six strain curves for six different LA segments in each view (four- and two-chamber view), which means that 12 LA segments were explored in each subject. However, in order to facilitate the calculation of longitudinal strain, the software provides one curve, which represents an average longitudinal strain of six segments in four- and two-chamber views. This curve represented total LA longitudinal strain—the parameter of LA reservoir function. LA total longitudinal strain and corresponding strain rates were calculated by averaging all the values obtained in four- and two-chamber apical views. The LA systolic strain rate was measured at LV systolic phase and it shows reservoir function, while early and late LA diastolic strain rates were measured during early LV filling and throughout late LV diastolic phase, showing conduit and booster pump functions respectively. A cardiologist (MT) performed LA strain analysis and this investigator was blinded for the fact which subject was the part of the cancer or control group. Figure 2 shows the methodology of LA longitudinal strain evaluation and Figure 3 represents the evaluation of LA strain rates in the present study.

The data were analyzed by using SPSS version 21 (SPSS, Inc, Chicago, IL, USA). Continuous variables were presented as mean ± standard deviation (SD). Normal distribution of included variables was confirmed by the Kolmogorov–Smirnov test. The Student’s *t*-test was used to detect differences between the two groups. LAVs and LA longitudinal strain was also presented with median values and interquartile ranges. The interquartile range was the range between the first and third quartile. The differences in proportions were compared by using the χ². The cut-off values for LA total longitudinal strain (< |–27%|) were used from the literature [11]. The association between age, gender, BMI, hypertension, diabetes, and presence of cancer with reduced total LA longitudinal strain was determined by the univariate logistic regression analysis (odds ration—OR and 95% confidence interval—CI) in the whole study population. The multivariate logistic regression analysis included all the mentioned parameters. The *p*-value < 0.05 was considered statistically significant.

## 3. Results

Age, gender distribution, BMI, blood pressure, prevalence of hypertension, diabetes, and smoking was similar between the controls and the cancer patients (Table 1). The study included 14 females with gynecological cancers (8 ovary and 6 uterus), 15 women with breast cancer, 38 patients with gastrointestinal cancers (12 esophagus, 8 gastric, 3 gallbladder, 13 pancreas, and 2 colon), 10 patients with sarcoma, and 15 subjects with lung cancer (Table 1).

### 3.1. Left Ventricular Structure and Function

There was no difference in LV diameters, wall thickness, and ejection fraction between the controls and the cancer group (Table 2). LV mass index and the parameters of LV diastolic function (E/A and E/e’ ratio) were similar between the observed groups (Table 2). 

### 3.2. Left Atrial Phasic Function

Minimal and pre-A LAVs indexed for BSA were significantly higher in the cancer patients with the borderline significance only for maximal LAVI (Table 3). Consequently, total and passive LAEF were significantly lower in the cancer patients, whereas active LAEF was significantly higher in this group of patients (Table 3). This illustrates that all LA reservoir and conduit functions, assessed with LA volumetric analysis, were deteriorated in the cancer group. On the other hand, LA pump function was elevated in the cancer group in comparison with the controls (Table 3).

LA total longitudinal strain was significantly lower in the cancer patients than in the controls, which confirms the impairment of LA reservoir function in these patients (Table 3). Further strain analysis of LA phasic function using strain rates showed that LA function during systole and early diastole was reduced in the cancer group, whereas it was increased during late diastole (Table 3).

### 3.3. Univariate and Multivariate Logistic Regression Analysis

LV hypertrophy, hypertension, diabetes, and cancer were each associated with reduced LA strain in the univariate analysis, but in the multivariate analysis diabetes only LV hypertrophy, cancer, and hypertension were independently associated with decreased LA longitudinal strain (Table 4).

## 4. Discussion

There are several findings of this study that deserve our attention and further discussion. First, minimal and pre-A LA indexed volumes were significantly higher in the cancer patients even before initiation of chemo- or radiotherapy. Second, reservoir and conduit LA functions were reduced in the cancer patients, whereas LA booster pump function was elevated in this group. This was showed by both volumetric and strain analysis of LA phasic function. Third, the presence of cancer by itself was associated with reduced LA longitudinal strain independently of other important demographic and clinical characteristics. 

Our study showed that minimal and pre-A LAVIs were significantly higher in the cancer patients with borderline statistical significance for maximal LAVI. Yaylali et al. showed that maximal and pre-contraction LAVs were significantly higher in the patients after anthracycline therapy in comparison with the healthy controls [7]. Shi et al. did not find any difference in 3DE LAVIs measured immediately after completion of anthracycline therapy in non-Hodgkin lymphoma patients and the controls [6]. On the other hand, Li et al. found significantly lower LAVIs in the long-term survivors of childhood cancers than in the control group [14]. Interestingly, Bergamini et al. showed that there was no difference in maximal LAVI between patients with and without cardiotoxicity [9]. 

The results regarding LA phasic function are not concomitant among different authors, but it seems that a kind of pattern does exist. Our findings showed that LA reservoir and conduit functions were reduced in the cancer patients, whereas LA pump function was elevated in the same group. This elevation of LA pump function is probably a compensatory mechanism that serves to maintain LV filling volume during late diastole, which we described earlier [15,16,17]. However, LV filling pressure assessed with mitral E/e´ ratio was not increased in our population of cancer patients in comparison with the controls. Our hypothesis is that our patients were possibly examined too early to detect increased LV filling pressure, which might develop afterwards. Previous studies detected the existence of LV diastolic dysfunction in cancer patients very soon (7 days) after chemotherapy was started [2,18]. Therefore, the question that arises is whether LV diastolic dysfunction is only the consequence of anti-cancer therapy or cancer itself has its own negative impact on it. Perhaps increased LA pump function represents a compensatory mechanism to overcome increased LV filling pressure that will develop. Furthermore, one should not forget that Doppler-derived parameters (E/e’ and E/A) are not the only criteria that reflect increased LV filling pressure. Diastolic strain rates could also be used for this purpose, as it was done in the mentioned studies [2,18]. Both parameters, early and late diastolic strain rates, were impaired in our investigation. 

Shi and Yaylali reported significantly reduced passive LAEF and increased active LAEF, which demonstrated impaired conduit LA function and elevated booster pump LA function in breast cancer and non-Hodgkin lymphoma patients treated with anthracyclines [6,7]. Both groups of authors used only volumetric evaluation of LA phasic function. Li et al. did not find any difference between the patients who had chemotherapy long time ago and the controls using only volumetric assessment of LA phasic function. Interestingly, strain analysis of LA phasic function showed that LA pump function was significantly reduced among cancer survivors [14]. Patel et al. reported no statistically significant reduction in LA longitudinal strain among children and young adult survivors treated with anthracyclines [19]. However, the limited number of subjects included in this study might be the main reason for not reaching statistically important difference in LA longitudinal strain between participants before and after anthracycline therapy [19]. The same group of authors made an interesting comparison of their LA strain results with the previously published age-specific LA strain in children and young adults. Namely, LA longitudinal strain values in the cancer patients before chemotherapy gradually decreased with age, which was not the situation in the healthy population where LA longitudinal strain was very low among children (<10 years), significantly higher in puberty (11–17 years), and slightly decreased in young adulthood (17–20 years) [20]. Interestingly, LA longitudinal strain among chemotherapy-naive children and young adults with cancer was mainly higher than in the healthy young population.

An important finding of the present study is the relationship between cancer and LA dysfunction independent of common risk factors for LA remodeling such as age, obesity, hypertension, diabetes, LV hypertrophy, and LV diastolic function. The clinical importance of this finding lies in the fact that current knowledge of this topic indicates chemo- or radiotherapy as the main responsible factor for cardiac remodeling in the cancer patients. Our results show that cancer itself could be responsible for cardiac remodeling even before starting anti-cancer therapy, which underlines the necessity for more detailed evaluation of LA and overall cardiac function before initiation of anti-cancer therapy. Furthermore, LA dysfunction is associated with atrial fibrillation occurrence which is more frequent arrhythmia in the cancer patients [20]. Therefore, it is of great importance to timely identify cancer patients who have discrete signs of cardiac remodeling at baseline in order to prevent development of cardiotoxicity and cardiac arrhythmias.

There are several potential mechanisms that could explain the independent association between cancer and impaired LA phasic function. Numerous circulating biomarkers, cytokines, vasoactive peptides, pro-BNP, and pro-ANP that are significantly increased in cancer patients could explain cancer-induced cardiotoxicity and eventually LA remodeling [21]. Our study was retrospective and included patients did not have any sign of heart failure, which is why biomarkers such as BNP or pro-BNP were not determined. Inflammation is also common for cancer and cardiac changes, which could also be the potential mechanisms that could explain our findings. Increased activation of bio-hormonal systems such as sympathetic nervous and renin-angiotensin-aldosterone systems, which is detected in cancer microenvironment [22,23], could be also responsible for LA remodeling in the these patients, as it was previously showed in patients with other pathologies [15,16,17,24]. Oncological patients significantly change their lifestyle and particularly reduce physical activity, which makes them susceptible to cardiac cachexia and other health issues that might contribute to LA remodeling, as is the situation with LV [25,26]. The prevalence of smokers in the cancer patients tended to be higher, suggesting that atherosclerosis or arterial stiffness might be more severe in this group, which might influence the alterations of LA phasic volumes and functions. 

The main strength of this study is the unique group of untreated cancer patients and findings that indicate impaired LA phasic function in oncology patients comparing with controls. These data might imply that chemo- and radiotherapy are not the only factors responsible for cardiac remodeling and cardiotoxicity, as it was believed previously. Additionally, this could help in determination of patients who are at higher risk of cardiotoxicity during anti-cancer therapy.

The present investigation has several limitations. The cancer group is not pathologically homogenous. However, our aim was not to investigate the effect of only one type of cancer. Additionally, as cardiologists in the clinical practice, we are not treating only one type of cancer, but all cancer patients. Important selection bias came from the fact that patients who are not receiving potentially cardiotoxic therapy (for example brain tumors) were not referred to echocardiohgraphic treatment and not included in this investigation. Furthermore, this was a retrospective study conducted at a tertiary center and the oncologists were only referring patients who potentially might receive cardiotoxic agents to the echocardiography department, which is why there could be a degree of selection bias present. The weight loss, which possibly occurred in the patients with cancer, was not considered as a confounding variable on LA remodeling and it should be included in future investigations. Asymptomatic atrial fibrillation could not be excluded as one of the potential reasons for deteriorated of LA function. LA evaluation was mostly performed on conventional echocardiographic views and not in views dedicated to LA assessment. We had to accept this limitation considering that the study was retrospective and there was no possibility to repeat the examination. This is a retrospective study and the causal relationship between cancer and LA phasic function could not be definitely established. On the other hand, it would be difficult to conduct a follow-up study with untreated cancer patients.

## 5. Conclusions 

Our study showed that LA phasic function is significantly deteriorated in the cancer patients even before initiation of chemo- or radiotherapy. The volumetric and strain analysis provided concurrent results that showed significantly reduced LA reservoir and conduit functions, but compensatory increased LA booster pump function. The presence of cancer was independent of other demographic and clinical parameters associated with reduced LA total longitudinal strain. The question that arises is whether or not chemo- and radiotherapy actually do have such a negative impact on cardiac remodeling or if a large part of this remodeling could be ascribed to the cancer itself. Future studies with larger number of cancer patients are necessary to confirm the relationship between cancer and LA remodeling and its predictive value during chemo- and radiotherapy. 

## Figures and Tables

**Table 1:** Demographic characteristics and clinical parameters of study population

	Controls (n = 40)	Cancer (n = 92)	*p*
Age (years)	52 ± 7	54 ± 8	0.173
Female (%)	18 (45)	48 (52)	0.570
BMI (kg/m^2^)	26.5 ± 3.1	26.7 ± 3.3	0.745
Heart rate (beats/min)	67 ± 8	70 ± 9	0.071
Systolic blood pressure (mmHg)	132 ± 10	134 ± 11	0.326
Diastolic blood pressure (mmHg)	76 ± 7	78 ± 8	0.173
Diabetes (%)	4 (10)	9 (10)	1.00
Hypertension (%)	7 (18)	19 (21)	0.813
Smoking (%)	8 (20)	34 (37)	0.068
Cancer localization			
Gynecological (%) ^a^	-	14 (15)	-
Breast (%)	-	15 (16)	-
Gastrointestinal (%) ^b^	-	38 (41)	-
Sarcoma (%)	-	10 (11)	-
Lungs (%)	-	15 (16)	-

BMI—body mass index, BP—blood pressure, DM—type 2 diabetes mellitus, ^a^—ovary and uterus; ^b^—esophagus, gastric, gallbladder, pancreas, and colon.

**Table 2:** Echocardiographic parameters of left ventricular structure and function in the study population

	Controls (n = 40)	Cancer (n = 92)	*p*
LVEDD (mm)	47.7 ± 3.9	47.4 ± 3.7	0.674
LVESD (mm)	30.1 ± 3.3	30.4 ± 3.5	0.646
IVS (mm)	9.6 ± 1.0	9.8 ± 1.1	0.326
LVMI (g/m2)	78 ± 14	81 ± 16	0.306
EF (%)	63 ± 4	62 ± 4	0.189
E/A ratio	1.18 ± 0.25	1.11 ± 0.22	0.110
E/e´	8.4 ± 2.5	8.9 ± 2.7	0.320

A—late diastolic mitral flow (pulse Doppler); E—early diastolic mitral flow (pulsed Doppler); e’—average of the peak early diastolic relaxation velocity of the septal and lateral mitral annulus (tissue Doppler); EF—ejection fraction; IVS—interventricular septum; LVMI—left ventricular mass index; LVEDD—left ventricle end-diastolic dimension; LVESD—left ventricle end-systolic dimension.

**Table 3:** LA phasic function determined by volumetric and strain method in the study population

	Controls (n = 40)	Cancer (n = 92)	*p*
**LA volume analysis**			
LAV_max_/BSA (mL/m^2^)	26.8 ± 4.8	28.7 ± 5.2	0.051
LAV_min_/BSA (mL/m^2^)	12.1 ± 3.9	14.1 ± 4.5	0.016
LAV_pre-a_/BSA (mL/m^2^)	18.3 ± 5.5	22.7 ± 6.2	< 0.001
LA TotEF (%)	55 ± 7	51 ± 7	0.003
LA PassEF (%)	32 ± 6	21 ± 6	< 0.001
LA ActEF (%)	34 ± 7	38 ± 8	0.009
**LA mechanics**			
Total longitudinal strain (%)	38 ± 8	32 ± 7	< 0.001
Systolic strain rate (s^−1^)	1.81 ± 0.55	1.53 ± 0.45	0.003
Early diastolic strain rate (s^−1^)	−2.03 ± 0.67	−1.64 ± 0.55	< 0.001
Late diastolic strain rate (s^−1^)	−1.56 ± 0.51	−1.88 ± 0.66	0.007

**Table 4:** Univariate and multivariate logistic regression analysis

	Univariate			Multivariate		
	OR	95% CI	*p*	OR	95% CI	*p*
**Reduced LA longitudinal strain (<|–27%|)**						
Age (years)	1.11	0.88–1.33	0.208	1.03	0.85–2.34	0.241
Gender (M)	1.13	0.92–1.54	0.136	0.98	0.66–2.12	0.177
BMI (kg/m^2^)	1.48	0.73–4.68	0.349	1.25	0.72–6.53	0.239
E/e’ ratio	1.87	0.94–3.65	0.206	1.43	0.87–4.79	0.329
LV hypertrophy (Y/N)	2.93	1.45–7.16	0.003	2.27	1.87–10.20	0.018
Diabetes (Y/N)	1.23	1.05–1.46	0.038	1.14	0.83–8.67	0.314
Hypertension (Y/N)	2.45	1.25–5.37	< 0.001	2.06	1.10–10.13	0.012
Cancer (Y/N)	4.04	1.74–16.46	< 0.001	3.55	2.11–18.70	< 0.001

Y—yes, N—no; OR—odd ratio; CI—confidence interval.
